# (*E*)-2,3-Dimethyl-*N*-(2-nitro­benzyl­idene)aniline

**DOI:** 10.1107/S1600536810024165

**Published:** 2010-06-26

**Authors:** M. Nawaz Tahir, Muhammad Ilyas Tariq, Shahbaz Ahmad, Muhammad Sarfraz, Abdul Qayyum Ather

**Affiliations:** a Department of Physics, University of Sargodha, Sargodha, Pakistan; b Department of Chemistry, University of Sargodha, Sargodha, Pakistan; c Applied Chemistry Research Center, PCSIR Laboratories Complex, Lahore 54600, Pakistan

## Abstract

In the title compound, C_15_H_14_N_2_O_2_, the 2,3-dimethyl­anilinic and benzaldehyde groups are planar, with r.m.s. deviations of 0.0101 and 0.0241 Å, respectively, and are oriented at a dihedral angle of 11.69 (3)°. The nitro group is inclined to the benzaldehyde group by 34.02 (9)°. The mol­ecule adopts an *E* configuration about the C=N bond. In the crystal, mol­ecules are linked *via* C—H⋯O inter­actions, giving rise to the formation of zigzag polymeric chains extending along [010]. They are also linked by C—H⋯π, and π–π inter­actions [centroid–centroid distance of 3.7185 (11) Å] involving symmetry-related aniline and benzene rings. The H atoms of the *ortho*-methyl group are disordered over two sites with a refined occupancy ratio of 0.69 (2):0.31 (2).

## Related literature

For the crystal structures of similar compounds, see: Tahir *et al.* (2010 ▶); Tariq *et al.* (2010 ▶).
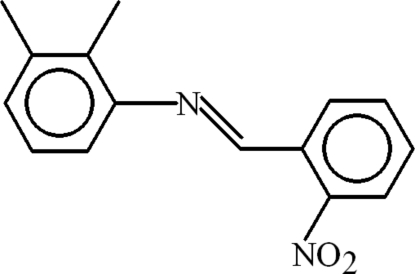


## Experimental

### 

#### Crystal data

C_15_H_14_N_2_O_2_
*M* *_r_* = 254.28
Monoclinic, 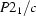

*a* = 12.2910 (6) Å
*b* = 15.1422 (9) Å
*c* = 7.3384 (3) Å
β = 107.091 (2)°
*V* = 1305.46 (11) Å^3^
*Z* = 4
Mo *K*α radiation
μ = 0.09 mm^−1^
*T* = 296 K
0.32 × 0.15 × 0.15 mm

#### Data collection

Bruker Kappa APEXII CCD diffractometer
Absorption correction: multi-scan (*SADABS*; Bruker, 2005 ▶) *T*_min_ = 0.985, *T*_max_ = 0.987
10220 measured reflections
2362 independent reflections
1705 reflections with *I* > 2σ(*I*)
*R*_int_ = 0.029

#### Refinement

*R*[*F* ^2^ > 2σ(*F* ^2^)] = 0.041
*wR*(*F* ^2^) = 0.111
*S* = 1.03
2362 reflections
171 parameters
H-atom parameters constrained
Δρ_max_ = 0.13 e Å^−3^
Δρ_min_ = −0.16 e Å^−3^

### 

Data collection: *APEX2* (Bruker, 2007 ▶); cell refinement: *SAINT* (Bruker, 2007 ▶); data reduction: *SAINT*; program(s) used to solve structure: *SHELXS97* (Sheldrick, 2008 ▶); program(s) used to refine structure: *SHELXL97* (Sheldrick, 2008 ▶); molecular graphics: *ORTEP-3 for Windows* (Farrugia, 1997 ▶) and *PLATON* (Spek, 2009 ▶); software used to prepare material for publication: *WinGX* (Farrugia, 1999 ▶) and *PLATON*.

## Supplementary Material

Crystal structure: contains datablocks global, I. DOI: 10.1107/S1600536810024165/su2187sup1.cif

Structure factors: contains datablocks I. DOI: 10.1107/S1600536810024165/su2187Isup2.hkl

Additional supplementary materials:  crystallographic information; 3D view; checkCIF report

## Figures and Tables

**Table 1:** Hydrogen-bond geometry (Å, °) *Cg*1 is the centroid of the C1–C6 ring.

*D*—H⋯*A*	*D*—H	H⋯*A*	*D*⋯*A*	*D*—H⋯*A*
C8—H8*A*⋯O2^i^	0.96	2.51	3.438 (2)	162.00
C8—H8*B*⋯*Cg*1^ii^	0.96	2.89	3.680 (2)	141

Symmetry codes: (i) 
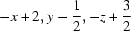
; (ii) 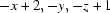.

**Fig. 1.**
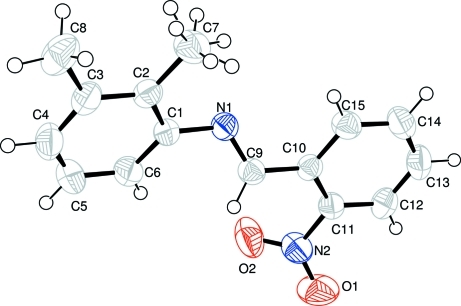
View of the molecular structuite of the title molecule, with the atom numbering scheme. The thermal ellipsoids are drawn at the 50% probability level. H-atoms are shown as small circles of arbitrary radii.

**Fig. 2.**
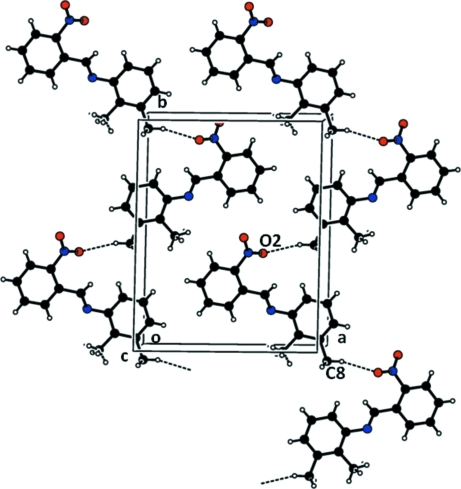
A partial crystal packing which shows that molecules form polymeric chains extending along [010].

## supplementary crystallographic information

### Comment

In continuation of our research on the synthesis and crystal structure analysis of various Schiff bases of 2,3-dimethylaniline (Tariq *et al.*, 2010; Tahir *et al.*, 2010), we report herein on the crystal structure of the title compound, where the nitro group is in the ortho position. This structure differs from that reported earlier (Tariq *et al.*, 2010) for 2,3-dimethyl-*N*-[(*E*)-4-nitrobenzylidene]aniline, where the nitro group is in the *para*-position.

In the title molecule (Fig. 1) the 2,3-dimethylaniline group A (C1—C8/N1) is planar, to within 0.0101 Å, and the benzylidene group B (C9—C15) is also planar, to within 0.0241 Å. The dihedral angle between mean planes A and B is 11.69 (3)°. The nitro group (O1/N2/O2) is oriented at 34.02 (9)° with respect to the mean plane of the parent group B. The molecule adopts an E configuration about the C1═ N9 bond, whose bond length is 1.263 (2) Å. The bond lengths are comparable with those in the structures cited above.

In the crystal structure the molecules are linked by C—H···O interactions to form zigzag polymeric chains extending along [010] (Table 1, Fig. 2). There also exist C-H···π interactions, and π–π interactions [centroid-to-centroid distance = 3.7185 (11) Å] between symmetry related aniline benzene rings (Table 1).

Footnote for Table 1: Cg1 is the centroid of benzene ring (C1-C6).

### Experimental

Equimolar quantities of 2,3-dimethylaniline and 2-nitrobenzaldehyde were refluxed in methanol for 45 min resulting in an orange solution. The solution was kept at RT and affoarded palepink rod-like crystals, suitable for X-ray diffraction analysis, after 24 h.

### Refinement

The H-atoms of the methyl group in the *ortho* position are disordered over two sites with a refined occupancy ratio of 0.69 (2):0.31 (2). All the H-atoms were positioned geometrically (C–H = 0.93, 0.96 Å) and refined as riding with *U*_iso_(H) = k × U_eq_(C), where k = 1.2 for aryl H-atoms and k = 1.5 for methyl H-atoms.

### Crystal data

**Table:**

C_15_H_14_N_2_O_2_	*F*(000) = 536
*M**_r_* = 254.28	*D*_x_ = 1.294 Mg m^−^^3^
Monoclinic, *P*2_1_/*c*	Mo *K*α radiation, λ = 0.71073 Å
Hall symbol: -P 2ybc	Cell parameters from 1705 reflections
*a* = 12.2910 (6) Å	θ = 2.2–25.3°
*b* = 15.1422 (9) Å	µ = 0.09 mm^−^^1^
*c* = 7.3384 (3) Å	*T* = 296 K
β = 107.091 (2)°	Rod, pale pink
*V* = 1305.46 (11) Å^3^	0.32 × 0.15 × 0.15 mm
*Z* = 4	

### Data collection

**Table:**

Bruker Kappa APEXII CCD diffractometer	2362 independent reflections
Radiation source: fine-focus sealed tube	1705 reflections with *I* > 2σ(*I*)
graphite	*R*_int_ = 0.029
Detector resolution: 8.10 pixels mm^-1^	θ_max_ = 25.3°, θ_min_ = 2.2°
ω scans	*h* = −14→14
Absorption correction: multi-scan (*SADABS*; Bruker, 2005)	*k* = −18→18
*T*_min_ = 0.985, *T*_max_ = 0.987	*l* = −5→8
10220 measured reflections	

### Refinement

**Table:**

Refinement on *F*^2^	Primary atom site location: structure-invariant direct methods
Least-squares matrix: full	Secondary atom site location: difference Fourier map
*R*[*F*^2^ > 2σ(*F*^2^)] = 0.041	Hydrogen site location: inferred from neighbouring sites
*wR*(*F*^2^) = 0.111	H-atom parameters constrained
*S* = 1.02	*w* = 1/[σ^2^(*F*_o_^2^) + (0.0511*P*)^2^ + 0.2309*P*] where *P* = (*F*_o_^2^ + 2*F*_c_^2^)/3
2362 reflections	(Δ/σ)_max_ < 0.001
171 parameters	Δρ_max_ = 0.13 e Å^−^^3^
0 restraints	Δρ_min_ = −0.16 e Å^−^^3^

### Special details

**Table:**

Geometry. Bond distances, angles *etc*. have been calculated using the rounded fractional coordinates. All su's are estimated from the variances of the (full) variance-covariance matrix. The cell e.s.d.'s are taken into account in the estimation of distances, angles and torsion angles
Refinement. Refinement of *F*^2^ against ALL reflections. The weighted *R*-factor *wR* and goodness of fit *S* are based on *F*^2^, conventional *R*-factors *R* are based on *F*, with *F* set to zero for negative *F*^2^. The threshold expression of *F*^2^ > σ(*F*^2^) is used only for calculating *R*-factors(gt) *etc*. and is not relevant to the choice of reflections for refinement. *R*-factors based on *F*^2^ are statistically about twice as large as those based on *F*, and *R*- factors based on ALL data will be even larger.

### Fractional atomic coordinates and isotropic or equivalent isotropic displacement parameters (Å^2^)

**Table:**

	*x*	*y*	*z*	*U*_iso_*/*U*_eq_	Occ. (<1)
O1	0.57216 (14)	0.48517 (10)	0.7186 (2)	0.0898 (6)	
O2	0.66972 (12)	0.41328 (10)	0.5671 (3)	0.0865 (7)	
N1	0.72326 (11)	0.16848 (9)	0.60756 (19)	0.0440 (4)	
N2	0.59423 (13)	0.41809 (10)	0.6438 (2)	0.0602 (6)	
C1	0.84205 (12)	0.15240 (10)	0.6480 (2)	0.0411 (5)	
C2	0.88063 (13)	0.06562 (10)	0.6889 (2)	0.0432 (5)	
C3	0.99754 (14)	0.04808 (12)	0.7314 (2)	0.0508 (6)	
C4	1.07104 (15)	0.11659 (15)	0.7281 (3)	0.0629 (7)	
C5	1.03219 (15)	0.20131 (14)	0.6825 (3)	0.0670 (8)	
C6	0.91756 (14)	0.21936 (12)	0.6413 (3)	0.0541 (6)	
C7	0.79775 (11)	−0.00684 (9)	0.6917 (3)	0.0630 (7)	
C8	1.04291 (11)	−0.04434 (9)	0.7794 (3)	0.0704 (7)	
C9	0.69105 (12)	0.23778 (11)	0.6723 (2)	0.0423 (5)	
C10	0.56911 (12)	0.25563 (10)	0.6417 (2)	0.0398 (5)	
C11	0.52334 (13)	0.34016 (11)	0.6387 (2)	0.0444 (5)	
C12	0.41029 (14)	0.35494 (13)	0.6243 (3)	0.0556 (6)	
C13	0.33943 (15)	0.28376 (14)	0.6115 (3)	0.0609 (7)	
C14	0.38193 (15)	0.19944 (13)	0.6158 (3)	0.0596 (7)	
C15	0.49477 (14)	0.18559 (11)	0.6304 (2)	0.0501 (6)	
H4	1.14863	0.10520	0.75744	0.0755*	
H5	1.08312	0.24631	0.67951	0.0804*	
H6	0.89095	0.27645	0.60905	0.0649*	
H7A	0.79909	−0.01850	0.82093	0.0945*	0.69 (2)
H7B	0.81863	−0.05936	0.63677	0.0945*	0.69 (2)
H7C	0.72254	0.01102	0.61921	0.0945*	0.69 (2)
H8A	1.12430	−0.04385	0.80782	0.1056*	
H8B	1.01086	−0.08255	0.67275	0.1056*	
H8C	1.02244	−0.06541	0.88832	0.1056*	
H9	0.74495	0.27808	0.74006	0.0507*	
H12	0.38266	0.41218	0.62325	0.0667*	
H13	0.26279	0.29245	0.59991	0.0730*	
H14	0.33385	0.15133	0.60884	0.0714*	
H15	0.52174	0.12810	0.63275	0.0601*	
H7D	0.74650	0.01276	0.75999	0.0945*	0.31 (2)
H7E	0.83851	−0.05791	0.75358	0.0945*	0.31 (2)
H7F	0.75525	−0.02168	0.56333	0.0945*	0.31 (2)

### Atomic displacement parameters (Å^2^)

**Table:**

	*U*^11^	*U*^22^	*U*^33^	*U*^12^	*U*^13^	*U*^23^
O1	0.0974 (12)	0.0457 (8)	0.1170 (13)	0.0068 (8)	0.0169 (9)	−0.0163 (8)
O2	0.0608 (9)	0.0664 (10)	0.1409 (15)	−0.0066 (7)	0.0428 (9)	0.0129 (9)
N1	0.0396 (7)	0.0394 (8)	0.0527 (8)	0.0020 (6)	0.0129 (6)	−0.0001 (6)
N2	0.0526 (9)	0.0436 (9)	0.0793 (11)	0.0034 (7)	0.0114 (8)	0.0045 (8)
C1	0.0369 (8)	0.0433 (9)	0.0425 (8)	0.0001 (7)	0.0109 (7)	−0.0041 (7)
C2	0.0447 (9)	0.0447 (10)	0.0398 (8)	0.0039 (7)	0.0117 (7)	−0.0027 (7)
C3	0.0464 (9)	0.0624 (11)	0.0427 (9)	0.0125 (8)	0.0119 (7)	−0.0040 (8)
C4	0.0397 (9)	0.0864 (15)	0.0628 (12)	0.0059 (10)	0.0152 (8)	−0.0087 (10)
C5	0.0492 (11)	0.0747 (14)	0.0821 (14)	−0.0164 (10)	0.0270 (9)	−0.0100 (11)
C6	0.0517 (10)	0.0456 (10)	0.0680 (11)	−0.0040 (8)	0.0222 (8)	−0.0034 (8)
C7	0.0612 (11)	0.0442 (11)	0.0811 (13)	−0.0018 (9)	0.0170 (10)	0.0018 (9)
C8	0.0676 (12)	0.0758 (14)	0.0636 (12)	0.0317 (11)	0.0127 (9)	0.0010 (10)
C9	0.0399 (9)	0.0414 (9)	0.0434 (9)	−0.0004 (7)	0.0088 (7)	−0.0006 (7)
C10	0.0394 (8)	0.0431 (9)	0.0367 (8)	0.0030 (7)	0.0108 (6)	−0.0007 (7)
C11	0.0425 (9)	0.0448 (9)	0.0463 (9)	−0.0001 (7)	0.0137 (7)	−0.0002 (7)
C12	0.0480 (10)	0.0585 (11)	0.0634 (11)	0.0117 (9)	0.0213 (8)	−0.0006 (9)
C13	0.0405 (9)	0.0798 (15)	0.0660 (12)	0.0026 (9)	0.0215 (8)	0.0020 (10)
C14	0.0493 (10)	0.0675 (13)	0.0632 (12)	−0.0134 (9)	0.0186 (9)	0.0005 (10)
C15	0.0505 (10)	0.0440 (10)	0.0547 (10)	−0.0020 (8)	0.0138 (8)	−0.0002 (8)

### Geometric parameters (Å, °)

**Table:**

O1—N2	1.222 (2)	C14—C15	1.375 (3)
O2—N2	1.221 (2)	C4—H4	0.9300
N1—C1	1.423 (2)	C5—H5	0.9300
N1—C9	1.263 (2)	C6—H6	0.9300
N2—C11	1.461 (2)	C7—H7A	0.9600
C1—C2	1.399 (2)	C7—H7B	0.9600
C1—C6	1.385 (2)	C7—H7C	0.9600
C2—C3	1.403 (2)	C7—H7D	0.9600
C2—C7	1.501 (2)	C7—H7E	0.9600
C3—C4	1.381 (3)	C7—H7F	0.9600
C3—C8	1.509 (2)	C8—H8A	0.9600
C4—C5	1.375 (3)	C8—H8B	0.9600
C5—C6	1.379 (3)	C8—H8C	0.9600
C9—C10	1.474 (2)	C9—H9	0.9300
C10—C11	1.396 (2)	C12—H12	0.9300
C10—C15	1.386 (2)	C13—H13	0.9300
C11—C12	1.381 (2)	C14—H14	0.9300
C12—C13	1.372 (3)	C15—H15	0.9300
C13—C14	1.376 (3)		
			
O1···C15^i^	3.413 (2)	H6···C8^iv^	2.8800
O1···N2^ii^	3.194 (2)	H7B···C8	2.6600
O1···O1^ii^	3.209 (2)	H7B···H8B	2.3300
O2···C9	2.758 (2)	H7B···H14^xi^	2.5900
O1···H15^i^	2.8200	H7C···N1	2.3900
O1···H14^i^	2.9000	H7D···O2^iii^	2.9100
O1···H7F^iii^	2.9000	H7D···N1	2.5900
O1···H12	2.4900	H7D···H12^vi^	2.5200
O2···H9	2.4400	H7E···H8C	2.1900
O2···H8A^iv^	2.5100	H7E···H8B	2.3900
O2···H7D^v^	2.9100	H7E···C8	2.4700
N1···C9^v^	3.410 (2)	H7F···H14^xi^	2.4200
N2···O1^ii^	3.194 (2)	H7F···O1^v^	2.9000
N1···H7D	2.5900	H7F···N1	2.9400
N1···H7F	2.9400	H8A···H4	2.3200
N1···H15	2.6100	H8A···O2^ix^	2.5100
N1···H9^v^	2.9000	H8B···C7	2.9000
N1···H7C	2.3900	H8B···C3^viii^	2.9800
N2···H9	2.7700	H8B···C4^viii^	2.8600
C9···N1^iii^	3.410 (2)	H8B···C5^viii^	3.0800
C9···O2	2.758 (2)	H8B···H7B	2.3300
C13···C14^iii^	3.591 (3)	H8B···H7E	2.3900
C14···C13^v^	3.591 (3)	H8C···C7	2.8600
C15···O1^vi^	3.413 (2)	H8C···C3^vii^	2.8800
C1···H9^v^	3.0700	H8C···H7E	2.1900
C2···H8C^vii^	2.9800	H8C···C2^vii^	2.9800
C3···H8C^vii^	2.8800	H9···N2	2.7700
C3···H8B^viii^	2.9800	H9···C6	2.5900
C4···H8B^viii^	2.8600	H9···H6	2.2700
C5···H8B^viii^	3.0800	H9···N1^iii^	2.9000
C6···H9^v^	3.0800	H9···C1^iii^	3.0700
C6···H9	2.5900	H9···C6^iii^	3.0800
C7···H8B	2.9000	H9···O2	2.4400
C7···H8C	2.8600	H12···H7D^i^	2.5200
C8···H7B	2.6600	H12···O1	2.4900
C8···H6^ix^	2.8800	H13···H5^xii^	2.5400
C8···H7E	2.4700	H14···H7B^xi^	2.5900
C9···H6	2.7000	H14···O1^vi^	2.9000
H4···H8A	2.3200	H14···H7F^xi^	2.4200
H5···H13^x^	2.5400	H15···O1^vi^	2.8200
H6···C9	2.7000	H15···N1	2.6100
H6···H9	2.2700		
			
C1—N1—C9	118.67 (14)	C1—C6—H6	120.00
O1—N2—O2	123.76 (17)	C5—C6—H6	120.00
O1—N2—C11	118.25 (16)	C2—C7—H7A	109.00
O2—N2—C11	117.94 (15)	C2—C7—H7B	109.00
N1—C1—C2	117.89 (14)	C2—C7—H7C	109.00
N1—C1—C6	121.64 (14)	C2—C7—H7D	109.00
C2—C1—C6	120.44 (15)	C2—C7—H7E	109.00
C1—C2—C3	119.06 (15)	C2—C7—H7F	109.00
C1—C2—C7	120.05 (14)	H7A—C7—H7B	109.00
C3—C2—C7	120.88 (14)	H7A—C7—H7C	109.00
C2—C3—C4	119.11 (17)	H7B—C7—H7C	109.00
C2—C3—C8	120.75 (15)	H7D—C7—H7E	109.00
C4—C3—C8	120.14 (16)	H7D—C7—H7F	109.00
C3—C4—C5	121.53 (18)	H7E—C7—H7F	109.00
C4—C5—C6	119.84 (19)	C3—C8—H8A	109.00
C1—C6—C5	119.95 (17)	C3—C8—H8B	109.00
N1—C9—C10	120.82 (14)	C3—C8—H8C	109.00
C9—C10—C11	123.90 (14)	H8A—C8—H8B	109.00
C9—C10—C15	119.45 (14)	H8A—C8—H8C	109.00
C11—C10—C15	116.43 (15)	H8B—C8—H8C	109.00
N2—C11—C10	120.39 (15)	N1—C9—H9	120.00
N2—C11—C12	116.77 (15)	C10—C9—H9	120.00
C10—C11—C12	122.81 (16)	C11—C12—H12	121.00
C11—C12—C13	118.85 (18)	C13—C12—H12	121.00
C12—C13—C14	119.89 (18)	C12—C13—H13	120.00
C13—C14—C15	120.70 (18)	C14—C13—H13	120.00
C10—C15—C14	121.32 (16)	C13—C14—H14	120.00
C3—C4—H4	119.00	C15—C14—H14	120.00
C5—C4—H4	119.00	C10—C15—H15	119.00
C4—C5—H5	120.00	C14—C15—H15	119.00
C6—C5—H5	120.00		
			
C9—N1—C1—C2	140.61 (15)	C2—C3—C4—C5	−0.6 (3)
C9—N1—C1—C6	−41.5 (2)	C8—C3—C4—C5	179.15 (18)
C1—N1—C9—C10	−177.30 (13)	C3—C4—C5—C6	0.9 (3)
O1—N2—C11—C10	−149.86 (15)	C4—C5—C6—C1	0.7 (3)
O1—N2—C11—C12	32.3 (2)	N1—C9—C10—C11	−153.88 (15)
O2—N2—C11—C10	32.6 (2)	N1—C9—C10—C15	31.7 (2)
O2—N2—C11—C12	−145.21 (18)	C9—C10—C11—N2	7.4 (2)
N1—C1—C2—C3	−179.29 (13)	C9—C10—C11—C12	−174.92 (16)
N1—C1—C2—C7	−0.8 (2)	C15—C10—C11—N2	−178.04 (13)
C6—C1—C2—C3	2.8 (2)	C15—C10—C11—C12	−0.4 (2)
C6—C1—C2—C7	−178.69 (16)	C9—C10—C15—C14	175.24 (15)
N1—C1—C6—C5	179.62 (17)	C11—C10—C15—C14	0.4 (2)
C2—C1—C6—C5	−2.6 (3)	N2—C11—C12—C13	177.48 (17)
C1—C2—C3—C4	−1.2 (2)	C10—C11—C12—C13	−0.3 (3)
C1—C2—C3—C8	179.01 (15)	C11—C12—C13—C14	0.9 (3)
C7—C2—C3—C4	−179.72 (16)	C12—C13—C14—C15	−0.8 (3)
C7—C2—C3—C8	0.5 (2)	C13—C14—C15—C10	0.2 (3)

Symmetry codes: (i) −*x*+1, *y*+1/2, −*z*+3/2; (ii) −*x*+1, −*y*+1, −*z*+1; (iii) *x*, −*y*+1/2, *z*+1/2; (iv) −*x*+2, *y*+1/2, −*z*+3/2; (v) *x*, −*y*+1/2, *z*−1/2; (vi) −*x*+1, *y*−1/2, −*z*+3/2; (vii) −*x*+2, −*y*, −*z*+2; (viii) −*x*+2, −*y*, −*z*+1; (ix) −*x*+2, *y*−1/2, −*z*+3/2; (x) *x*+1, *y*, *z*; (xi) −*x*+1, −*y*, −*z*+1; (xii) *x*−1, *y*, *z*.

### Hydrogen-bond geometry (Å, °)

**Table:**

Cg1 is the centroid of the C1–C6 ring.

**Table:**

*D*—H···*A*	*D*—H	H···*A*	*D*···*A*	*D*—H···*A*
C8—H8A···O2^ix^	0.96	2.51	3.438 (2)	162.00
C8—H8B···Cg1^viii^	0.96	2.89	3.680 (2)	141

Symmetry codes: (ix) −*x*+2, *y*−1/2, −*z*+3/2; (viii) −*x*+2, −*y*, −*z*+1.
